# Rearing hogs on pasture minimally impacts pork composition

**DOI:** 10.1093/tas/txae114

**Published:** 2024-07-25

**Authors:** Chelsea Becker, Jonathan Campbell, Kathy Soder, Elizabeth A Hines

**Affiliations:** Department of Animal Science, Pennsylvania State University, University Park, PA 16802, USA; Department of Animal Science, Pennsylvania State University, University Park, PA 16802, USA; USDA-ARS, Pasture Systems and Watershed Management Research Unit, University Park, PA 16802, USA; Department of Animal Science, Pennsylvania State University, University Park, PA 16802, USA

**Keywords:** carcass, growth, pasture, pigs, pork

## Abstract

Managing swine on pasture is increasing in popularity for both the consumer and producer. This interest appears to be driven by an effort to create an improved perception of environmentally sustainable practices and increased animal welfare, while keeping start-up costs low. However, evidence-based guidance on pasture management practices that support quality pork production and environmentally sustainable procedures is lacking. The objective of this work was to quantify the impact of pasture rearing on pig growth efficiency and pork quality. In this pilot study, 20 pigs similar in genetics, age, weight, and sex ratio were divided across indoor (*n* = 10) and Outdoor (*n* = 10) housing environments. Pigs were weighed every 14 d and harvested upon reaching an average weight of 113 kg. Average starting body weights were similar between both groups (*P* = 0.98). Carcass quality was evaluated by measuring pH, loin eye area (LEA), back fat (BF) thickness, subjective color and marbling scores, and colorimetry (CIE color space [L*, a*, b*]) at the 10th rib. Final body weights at slaughter also showed no significant variation between housing groups (*P* = 0.98). No differences were observed in pork quality: pH 0 h (*P* = 0.53), 6 h (*P* = 0.29), 12 h (*P* = 0.80), and 24 h (*P* = 0.07) postmortem, LEA (*P* = 0.44), color (*P* = 0.73), and marbling (*P* = 0.40). However, hogs raised indoors had an increase in BF thickness (*P* = 0.04). Based on this pilot study, outdoor rearing conditions did not have significant impacts on pork quality. Further research will help to determine the impact that rearing scheme has on pH and BF.

## Teaser

The environment in which pigs are raised, whether indoors or outdoors, is colloquially believed to impact the quality of pork. Results of this study indicate that outdoor rearing conditions do not significantly affect pork quality, challenging conventional beliefs.

## Introduction

Pasture-based management practices are increasing in popularity among both consumers and producers. Consumers are searching for products and producers that meet their moral and ethical standards, such as those with high environmental sustainability goals (Gentry and McGlone, 2003), and the ability to support a pig’s natural behavior and environment (Lebret, 2008; Sørensen and Schrader, 2019). Producers are often willing to meet these standards set by consumers because it allows them to gain higher revenues through niche markets (Norwood and Lusk, 2011; Weiss, 2011). These specialty markets, paired with the potential to have low capital investments, have helped contribute to the overall interest in pasture-based management systems (Maiorano et al., 2013). However, producers who wish to pursue or expand a pastured pork business face challenges and struggle to find recommended best practices due to a lack of repeatable, science-based management techniques, particularly as it relates to the impact of pastured rearing conditions on meat production and quality.

The impact of pasture rearing on slaughter weights has been observed to vary (Gentry et al., 2002a; Hoffman et al., 2003), as well as differing pork quality attributes such as backfat (BF) thickness, loin eye area (LEA), marbling, and color when raising pigs on pasture (Gentry et al., 2002b; Pugliese et al., 2003; Maiorano et al., 2013). This variation in growth and pork quality makes it difficult for producers to establish best management practices (BMPs) that ensure pork quality and the continual improvement of production. Previous comparison studies have shown a large amount of variation in study methods and results; thus, this study was conducted to construct a direct comparison of indoor and outdoor housing and their impact on pork quality.

## Materials and Methods

### Animal Rearing

All animal procedures were approved by the Animal Care and Use Committee at The Pennsylvania State University (IACUC#: PROTO202101910). Twenty (*n* = 20) crossbred Yorkshire pigs (White Walker [Top Cut Genetics, LLC, IN] by Yorkshire-Cross sow [Penn State Herd]) were divided across 2 rearing conditions: indoor (*n* = 10), and outdoor (*n* = 10). There were 5 males and 5 females in each group. Pigs were placed into one of the two groups according to their body weights (indoor: 46.2 kg; outdoor: 46.1 kg; *P* = 0.98) to minimize variation at 14 wk of age. The Outdoor group was given unrestricted pasture access on approximately 0.13 ha on Penn State’s swine facilities with access to a customized wooden hutch, complete with straw bedding and a tin roof for shade. Pastures were rotated every four weeks. The Indoor group was housed on solid concrete flooring and pigs were placed into a single pen, sized at 14.12 m^2^ in a fully enclosed, naturally ventilated swine barn. Both groups were managed by the same personnel and fed a standardized grow–finish ration (Table 1) via a Smidley flap top feeder (Smidley, Britt, IA) ad libitum and given free access to clean water via an Edstrom nipple drinker system (Avidity Science, Waterford, WI). Rearing conditions were on the same site, within 267 m between the barn and the furthest outdoor lot used for rearing pigs.

**Table 1.** Feed nutrition labels for diets provided ad libitum to pigs on test^1^

	Grower 1	Grower 2	Grower finisher
Ingredient, %	Fed to hogs 60 to 100 lb	Fed to hogs 100 to 150 lb	Fed to hogs 150 to 250 lb
Crude protein	19.85	17.36	15.1
Calcium	0.62	0.6	0.52
Phosphorus	Total 0.54 / available 0.27	Total 0.52 / available 0.27	Total 0.47 / available 0.23
Sodium	0.18	0.18	0.18
Salt	0.35	0.35	0.35
Moisture	10.75	10.6	10.47
Oil	5.29	4.91	4.55
Ingredient, digestible			
Lysine	1.04	0.89	0.75
Threonine/lysine	0.64	0.65	0.67
Tryptophan/lysine	0.21	0.21	0.2
MC/lysine	0.47	0.51	0.55

^1^Values reflect information on dietary feed labels. Diets created utilizing a concentrate mixed in corn and soybean oil, a common approach to diet mixing and management in small farm and pastured settings for pigs. Pigs were given ad libitum access to feed and diets were transitioned based on average weight of the pigs for the duration of the project.

### Data Collection Methods

Individual body weights were collected at the beginning of the study and then every two weeks for fourteen consecutive weeks using an electronic Waypig scale (Farmer Boy Ag, Myerstown, PA) with a Digi-Star scale head (Fort Atkinson, WI). When pigs reached a target weight of 113 kg, each was sent to slaughter at the Penn State Meat Lab on a weekly schedule for collection of carcass and pork quality data. Color and marbling scores were assigned subjectively by one observer (Pork Quality Standards chart; National Pork Board, Des Moines, IA), and objectively using a Minolta colorimeter (CR—300; Konica Minolta, Wayne, NJ) that assigns values associated with color and light scores in the CIE color space: lightness (L*), redness (a*), and yellowness (b*). BF measurements were obtained in inches using a pork backfat ruler (Nasco Education, Fort Atkinson, WI), and LEA (in^2^) was calculated using cross-sectional measurements obtained from a plastic grid (Iowa State University Extension, Des Moines, IA). Post-slaughter pH was measured at 0-, 6-, 12-, and 24-h postmortem using a portable meat pH meter (HI 99161; Hanna Instruments, Woonsocket, RI). All carcass-quality components were collected at the 10th rib.

### Statistical Analyses

All statistical values were obtained using the PROC MIXED procedure of SAS 9.1 (Cary, NC), with individual pigs as the experimental unit and the pen housing (indoor vs. outdoor) as the fixed effect for growth and pork quality data. All analyses were considered statistically significant at *P* ≤ 0.05.

## Results

### Growth Was Not Impacted by Rearing Conditions

Starting body weights between both treatment groups were similar with the indoor group starting at an average weight of 46.2 kg and the outdoor having an average weight of 46.1 kg (*P* = 0.98; Table 2). Average daily gain (ADG) was lower for the Outdoor group during the first 4 wk of study (*P* < 0.01, *P* = 0.02), followed by another decrease at week 8 (*P* = 0.01). At all other sample times, there was no significant difference between the 2 treatment groups (week 6: *P* = 0.23; week 10: *P* = 0.75; week 12: *P* = 0.42; week 14: *P* = 0.21). Additionally, slaughter weights also showed no difference with the indoor group finishing at 125.4 kg and the outdoor group finishing at 118.8 kg (*P* = 0.08).

**Table 2.** Growth performance

Measurement taken at the 10th rib	Indoor^1^	Outdoor^2^	St. Error^3^	*P* value
Starting body weights, kg	45.98	46.05	1.84	0.98
Total average daily gain (ADG)	0.85^a^	0.75^b^	0.02	0.02
Slaughter weights, kg	125.35	118.79	5.69	0.08
Hot carcass weights, kg	95.88	92.12	1.78	0.15

^1^Twenty hogs housed solely indoors on concrete flooring.

^2^Twenty hogs housed on pasture.

^3^Average standard error for each variable.

^a,b^Superscript letters are used to represent significant differences between means.

### Pasture Housing Reduced Backfat on Market Hogs, But Did Not Influence Other Pork Quality Measures

Hot carcass weights showed no differences between the treatments with the Indoor group at 94.2 kg and the outdoor group at 93.8 kg (*P* = 0.13; Table 3). No differences in subjective color score (*P* = 0.73) or marbling score (*P* = 0.40) were observed between the two groups. Colorimeter values were also not statistically different: L* (*P* = 0.77), a* (*P* = 0.75), and b* (*P* = 0.71). Additionally, LEA values between the 2 groups were shown to have no difference (*P* = 0.44). However, backfat was greater (*P* = 0.04) for the indoor group at 0.89 cm, as compared to the outdoor group at 0.75 cm. Measurements taken for pH at 0 (*P* = 0.53), 6 (*P* = 0.29), 12 (*P* = 0.80), and 24 (*P* = 0.07) h postmortem showed a normal rate of decline with no statistical differences between the 2 groups for each of the sample times.

**Table 3.** Carcass and pork quality characteristics

Measurement taken at the 10th rib	Indoor^1^	Outdoor^2^	St. Error^3^	*P* value
Color score	2.61	2.56	0.29	0.73
Marbling score	1.44	1.75	0.23	0.40
Lightness (L*)	49.95	49.09	1.36	0.77
Redness (a*)	5.91	6.33	0.64	0.75
Yellowness (b*)	5.41	4.68	0.71	0.52
Loin eye area (LEA)	7.99	8.01	0.32	0.44
Backfat (BF)	0.89	0.75	0.06	0.05
pH at 0 h	6.02	5.93	0.15	0.53
pH at 6 h	5.45	5.53	0.07	0.29
pH at 12 h	5.42	5.43	0.04	0.80
pH at 24 h	5.40	5.48	0.42	0.07

^1^Twenty hogs housed solely indoors on concrete flooring.

^2^Twenty hogs housed on pasture.

^3^Average standard error for each variable.

^a,b^Superscript letters are used to represent significant differences between means.

## Discussion

Raising swine on pasture may benefit producers through decreased starting costs and increased potential revenue from niche markets (Norwood and Lusk, 2011). Previous studies on pastured pork quality have yielded conflicting results that may ultimately impact profit. Some studies have shown ADG increasing when pigs are reared on pasture during warmer months (Stern et al., 2003; Juska et al., 2013). Yet, there are others that show the opposite with the indoor groups surpassing ADG, regardless of environmental temperature as supported by the current pilot study (Hoffman et al., 2003; Hansen et al., 2006). However, there is still a wide range of studies that show no differences in ADG as related to rearing when both groups have been fed a corn-based diet (Gentry et al., 2002b; Maiorano et al., 2013).

Pork quality attributes are another source of variation in previous research studies, with color and marbling scores showing these vast differences. A few studies show that pigs reared indoors have higher subjective color and marbling scores, indicating a darker and more red-colored pork product with a higher percentage of intramuscular fat, regardless of birthing environment or diet (Gentry et al., 2002a, 2002b; Hansen et al., 2006). To support these findings, consumer studies have shown a preference for pork products that have higher marbling content and color scores when asked to evaluate on visual observation and at-home sensory evaluation (Norman et al., 2003; Moeller et al., 2010). Although, it is important to note that many pastured pork studies, including this pilot study, have shown no differences in either color or marbling scores (Gentry et al., 2002a; Stern et al., 2003). L*, a*, and b* values have also been shown to vary with many studies showing no differences between the two rearing environments when diet and genetics were considered (Stern et al., 2003; Hansen et al., 2006). Additional studies show indoor-reared pigs having higher colorimeter scores in all three values regardless of diet, space allowance, or genetics (Gentry et al., 2002b; Hoffman et al., 2003; Lebret et al., 2015).

Among pork quality attributes observed in this study as well as previous studies, BF seems to remain the most consistent. Multiple studies have shown that indoor-reared pigs have higher amounts of BF thickness regardless of housing conditions, time of year, or diet (Gentry et al., 2002b; Pugliese et al., 2003; Maiorano et al., 2013). However, there are still a few cases that show the opposite, with outdoor reared pigs developing more BF despite their access to a supplemental diet (Lebret, 2008; Dostálová et al., 2020). In relation to growth efficiency, BF is associated with reduced lean yield (Hoa et al., 2021). However, pork of high quality is considered to have a minimum of 1.78 cm of BF. Despite a higher BF, pigs in this study had similar LEA across treatments. Other studies that compared housing for growing pigs also saw no differences in LEA when pigs were given various diets and pasture access (Gentry et al., 2002b; Juska et al., 2013; Maiorano et al., 2013). Additionally, more studies are needed to explore the potential mechanisms and solutions for managing BF in pastured pigs.

Beyond carcass composition, ultimate pH is a strong predictor of pork quality. Extensive variation has been observed in pH across pastured pork studies. Most literatures, including this pilot study, do not indicate differences between the ultimate pH of pigs reared on pasture versus those raised in confinement when genetics, environment, and diet are considered (Stern et al., 2003; Juska et al., 2013; Maiorano et al., 2013). One study indicated that outdoor reared pigs had a higher ultimate pH when compared to indoor groups regardless of genetics or amount of pasture access (Lebret et al., 2015). The ideal ultimate pH for pork ranged between 5.7 and 6.1. It is important to note that the rate of pH decline has been shown to have a larger impact on similar pork quality attributes such as color and water holding capacity than the ultimate pH (Bidner et al., 2004; Kim et al., 2016). Both groups in the current study followed a steady rate of decline with pH values at all sampling times postmortem: 0 h (*P* = 0.53), 6 h (*P* = 0.29), 12 h (*P* = 0.80), and 24 h (*P* = 0.07).

Many factors play into these differences in pork quality; a few of those being genetics, diet, environment, and exercise (Edwards, 2005). Adjustment periods for pigs moved into outdoor environments also cause changes in growth and feed intake that could impact overall pork quality. The variables present between outdoor- and indoor-reared pigs are difficult to assess in research. However, in the current study, genetics and diet were controlled as a part of the experimental design. Further, feed was offered unrestricted to both groups of pigs; however, feed usage was not measured. Tracking feed usage by pigs across treatments might reveal additional influences on the differences observed in growth performance in this study. Additionally, pigs were slaughtered at a consistent weight, rather than a consistent time. This variation in days to slaughter allows for inconsistencies to potentially be observed in pork quality while also increasing the funds needed to raise these pigs to the ideal market weight.

## Conclusion

Enthusiasm for pasture-reared pork appears to be growing among both consumers and producers for perceived human health benefits, animal welfare concerns, environmental concerns, and economics. Yet, consistency of product and BMPs for both the pigs and environmental resources have not kept up with changing genetics and consumer trends. Standards for indoor pig production and meat quality already exist, thus comparison studies are necessary to develop management practices that improve consistency in pork products and allow recommendations for BMPs to producers that target pasture-based markets. Most studies completed in this area are highly variable in research parameters across treatments, while this research standardized genetics, location, and management to highlight the impact of indoor or outdoor housing systems. Although the results from this pilot study show that pork quality is minimally impacted by indoor or outdoor housing, we did observe an increase in BF thickness in pigs reared indoors. Additionally, there are indications that pasture-based housing had a limited effect on pH when pigs are of the same genetics and provided the same ration. The results of this pilot study indicate that further exploration into the impact of space, energy expenditure, or other environmental factors associated with pasture on pig performance and pork quality. Finally, pig growth did not seem to vary between housing groups. This pilot study data will support further research that can expand replicates into pens and include an assessment of the impact of pasture access on feed consumption.
